# Key drivers of hysterectomy among women of reproductive age in three states in India: comparative evidence from NFHS-4 and NFHS-5

**DOI:** 10.1186/s12905-024-02886-7

**Published:** 2024-02-09

**Authors:** Shri Kant Singh, Kirti Chauhan, Vrijesh Tripathi

**Affiliations:** 1 Department of Survey Research and Data Analytics, International Institute for Population Sciences, Mumbai, Maharashtra India; 2 Department of Biostatistics and Demography, International Institute for Population Sciences, Mumbai, Maharashtra India; 3 Department of Mathematics and Statistics, The University of the West Indies, St. Augustine, Trinidad and Tobago

**Keywords:** Hysterectomy, Andhra Pradesh, Telangana, Bihar, Decomposition, Multilevel analysis

## Abstract

**Purpose:**

According to the 4th and 5th rounds of National Family Health Survey (NFHS), there is high prevalence of hysterectomies in the three states of Andhra Pradesh Telangana and Bihar. The three said states have more than double the number of hysterectomies taking place than the national average. Our purpose is to analyse whether these rates are increasing, decreasing or have stabilized and their reasons thereof. Such an analyses will help the policy makers in recommending good clinical practices within their states.

**Material and methods:**

We used data from NFHS-4 (2015-16) and NFHS-5 (2019-2021) rounds. We calculated the differences in predicted probabilities for various factors, performed a Fairlie Decomposition analyses to quantify the positive and negative contributors in the prevalence of hysterectomy across the three states over two time points, and assessed the association of various socio-demographic characteristics to hysterectomy through a multilevel logistic regression model.

**Results and conclusion:**

The results show that out of a total of 80,976 eligible respondents from the states under study, 5826 respondents self-reported that they had a hysterectomy done. It was found that older age, living in rural areas, belonging to other backward classes and higher wealth quintile, and higher parity positively contributed to the increased prevalence of hysterectomies in the three states. Higher educational attainment and previous use of family planning methods acted as protective factors. Characteristics at the household level had the highest intra-class correlation value in the prevalence of hysterectomy among women, followed by the Primary Sampling Unit and District levels, indicating high clustering in the prevalence of hysterectomy at the household level in all three states. Heavy menstrual bleeding/pain was the leading cause of hysterectomies in all three states, followed by fibroids/cysts in Andhra Pradesh and Telangana and Uterine disorder/ prolapse in Bihar. Over 80% of hysterectomies took place in the private hospitals.

**Recommendations:**

The study recommends better, more efficient and accountable hysterectomy surveillance to ensure more sustainable woman’s reproductive health services in India. Government should adopt and implement standard regulatory guidelines to prevent provider-driven avoidable hysterectomies. Moreover, we recommend informing primary care professionals about the long-term health effects of hysterectomy and promoting alternate therapies for treating uterine fibroids and heavy bleeding.

**Supplementary Information:**

The online version contains supplementary material available at 10.1186/s12905-024-02886-7.

## Background

Hysterectomy is a surgical procedure among women in which the uterus is removed. Depending upon medical assessment, other portions of the female reproductive system, including ovaries, fallopian tubes, and cervix, may also be removed. Hysterectomy is the most common gynecological procedure performed in the United States of America (USA) [1]. It is generally estimated that hysterectomy rates are higher in high-income countries such as United States (26.2%), Australia (22.0%) and Ireland (22.2%) than in low-and-middle income settings such as in India where it is 11.35% [2–5]. For a number of years, this was seen as a matter of woman’s choice and control over her reproductive health matters. But there has been a growing interest in keeping the uterus due to hormonal benefits that go well beyond the child-bearing years. While hysterectomy may be the first line of treatment in cases of severe prolapse, cancer and postpartum bleeding, it can be avoided in cases of treatment of pain, heavy menstrual bleeding and presence of fibroids. It is suggested that the rate of surgical removal of the uterus is going down due to development of screening and preventive programmes and availability of minimally invasive techniques such as, myomectomy (a surgical method to remove only the fibroid), endometrial ablation devices, levonorgestrel intrauterine system (LNG-IUS) and uterine arterial embolization (which blocks the blood supply to the fibroids and causes them to shrink) [6–10]

While hysterectomy offers relief in the short-term for some cases, the surgery is not risk-free. Short-term morbidities include increased risk of intra-abdominal adhesions, postoperative infections, pelvic organ dysfunction, and thromboembolic events. A systematic review alongside several other studies highlight that long-term potential risks of hysterectomy which includes development of cardiovascular disease; hypertension and stroke; urinary tract cancer; thyroid cancer; incontinence; pelvic prolapse; pelvic organ fistula; lower urinary tract infection; ovarian failure and premature menopause, along with other consequences of estrogenic decline, including bone mineral density loss; vasomotor symptoms; frailty; depression; and a decline in cognitive function [11–13]. There is medical evidence that removing the uterus and ovaries results in considerable psychological and physiological harm [12, 13]. Hence, hysterectomy should be utilized only in cases where other treatment options have not provided desirable results.

Various community-based case studies within India have highlighted that women with no education but with health insurance are more vulnerable to unnecessary hysterectomies [14–16]. A study noted the prevalence of hysterectomy among women under 40 years of age was high in Andhra Pradesh (42%) and Telangana (47%) [17]. A case study in the Medak district of the then Andhra Pradesh, now Telangana, highlighted the involvement of medical insurance, gender bias, and a lack of ethical conduct in the medical field as major promoters of unnecessary hysterectomies in the area [16]. A cross-sectional study among rural women in Chittoor district, Andhra Pradesh, suggested that though hysterectomy was not common among women working in the public or private sectors, the prevalence was high, varying from 2.8% among housewives to 7.7% among women working as coolies, and 14.0% among women working in the agricultural sector [18]. Studies in a low-income setting in Gujarat’s Ahmedabad area found that hysterectomies were a primary cause of hospitalization and medical insurance claims [14]. It is noteworthy that women having low socioeconomic status have a substantially younger median age for hysterectomy, that is, less than 35 years [17, 18]. Thus, the inclusion of hysterectomies in health insurance schemes in the southern states is said to have contributed to a surge in these surgeries [19]. However, there was no data available whether a woman used her health insurance cover for the surgery.

In India, in the year 2013, a number of reports appeared in the press regarding “unwarranted” or “unnecessary” hysterectomies taking place in women as young as 29 years old [19, 20]. Unnecessary hysterectomy can be described as a medical condition that can be managed with an alternative treatment [4, 5]. The women were told that they might even develop cancer if they did not undergo the surgery [18, 21]. These led the Government of India to include questions regarding hysterectomy in the National Family Health Survey (NFHS)-4 since no national level figures were available [22]. The NFHS-4 and NFHS-5 have collected self-reported data on hysterectomies across the states and union territories in India. Case reports from Andhra Pradesh, Rajasthan and Chhattisgarh suggested that women belonging to the lower strata of society and working in unorganized sectors underwent hysterectomies in order to rid themselves of pain and/or bleeding and because they had completed their families [23]. Many of these women were very young and pre-menopausal at the time that they had hysterectomies. Most non-governmental organizations (NGOs) reported that the women underwent these surgeries in private clinics [15]. The costs of the operation plunged such households further into debt. A combination of factors such as financial incompetency, doctor’s preference, cultural taboos, a woman’s need to work on all days, a woman’s desire to lead a normal life and/or availability of government-sponsored health insurance schemes may influence the decision making process [24, 25]. Additionally, doctors commonly advise hysterectomy to women of reproductive age particularly from low-income households in India, suffering from excessive menstrual bleeding, fibroids, endometriosis, and uterine prolapse [26].

The National Health Family Survey (NFHS)-4 revealed that the prevalence of hysterectomy was 3% among women aged 15-49 years according to the study that covered 29 states and seven union territories [27]. The number of women, aged 15-49 years, undergoing hysterectomy varies from 2 to 63 per 1000 in different states of India [2]. A study done by Desai and her colleagues highlighted that approximately two-thirds of the hysterectomies were performed in private facilities, with a prevalence of 3.59% in the age group 30 to 39 years and 9.2% in the age group 40 to 49 years [28]. Factors associated with hysterectomy included higher age, parity (at least two children), not having had formal schooling, rural living, having had a previous cesarean section, and higher wealth status [28]. The analyses revealed that the prevalence of hysterectomies was particularly high in Andhra Pradesh, Telangana and Bihar.

## Rationale of the study

From NFHS-4 to NFHS-5, the prevalence of hysterectomy remained high in the three states namely, Andhra Pradesh, Telangana, and Bihar. It is known that the prevalence and major determinants may vary considerably by geographical location, socio-demographic and medical factors due to changes in uterine pathology health services, individual characteristics, and socio-cultural factors [2, 19, 27]. Health is a State subject, that is, all health services in India come under the purview of the State governments. It is hypothesized that more hysterectomies are taking place in these states due to better health infrastructure and more ‘effective’ implementation of health insurance schemes [2]. Equally, it could also be easily argued that more hysterectomies are occurring due to poor counselling, lack of medical facilities and/or due to poor state health infrastructure [27]. Therefore, we conducted a comparative analysis to know the key drivers of hysterectomies in the three states and whether there had been any increase or decrease in hysterectomies in these three states over the last two rounds of NFHS.

## Objectives

The present study addresses the following objectives:

1. To assess the difference in predicted probabilities of various factors contributing to hysterectomy from NFHS-4 (2015-16) to NFHS-5 (2019-21).
2. To investigate the variation in socio-demographic factors associated with hysterectomy among women aged 15-49 years in the three states by conducting multilevel logistic regression analyses of age, years of schooling, place of residence, religion, caste, number of children ever born, ever use of a family planning method and wealth index. We did not include women’s work status in our regression analyses because women perform very many functions within the house and on family land holdings for which they are not compensated monetarily. That is why a simple binary category between housewife and employed does not truly reflect either the amount, the need, or the nature and type of work that a particular woman does within her household.
3. To assess the trend in women seeking health services for the conduct of hysterectomies and the reasons for hysterectomies from NFHS-4 (2015-16) to NFHS-5 (2019-21). We also assessed the trend in inclusion of women in health insurance schemes.

## Data and methods

The current study is based on the Indian Demographic and Health Survey (DHS) rounds, i.e., NFHS-4 (2015-16), and NFHS-5 (2019-2021) conducted by the International Institute for Population Sciences, Mumbai [29, 30]. The NFHS sample is intended to provide national, state/union territory (UT), and district-level estimates of various indicators important for monitoring the sustainable development goals (SDGs) on population, health, nutrition, and gender equality. The sampling design of NFHS-5 has been developed using NFHS- 4 as the benchmark to provide population, health, and family welfare indicators’ estimates at district, State/UT, and national levels with reasonable precision. A stratified two-stage sampling design was used in the 707 districts’ rural and urban areas (as of 31st March 2017). Villages within each rural stratum were chosen from the sampling frame using probability proportional to size (PPS) with explicit stratification based on the Scheduled castes/ Scheduled tribes (SC/STs) percentage and female literacy. The households were chosen using a sampling frame created by mapping and listing households in all 707 districts’ primary sampling units (PSUs). NFHS employs four survey schedules—Household, Woman’s, Man’s, and Biomarker—that are administered in local languages via Computer Assisted Personal Interviewing (CAPI). The current study sample was derived from NFHS-4 which covered 601,509 households and 699,686 women aged 15–49 years (ever married sample only, which includes women who were divorced or separated or widowed) from 28,586 PSUs [31, 32] and NFHS-5 which covered 636,699 households with 724,115 eligible women aged 15-49 years (ever married) from 30,456 PSUs that comprised villages in rural areas and census enumeration blocks (CEBs) in urban areas [30].

### Outcome variable

Hysterectomy has been utilized as the outcome variable in this study. The NFHS-4 and NFHS-5 posed a series of questions to women regarding hysterectomy asking, “Have you ever had a procedure like this?” The answer was coded as “yes” and “no.” If yes, the women were asked more questions about the age at which hysterectomy was done, the location (public/private service centers), and the reason due to which the hysterectomies were done. Data was collected outlining reasons such as excessive menstrual bleeding, fibroids/cysts, uterine disorder, cancer, uterine prolapse, severe post-partum hemorrhage, cervical discharge, and other; and respondents could select more than one reason for having a hysterectomy. We also included the question if the respondents had health insurance.

### Predictor variables

Various predictor variables considered in the study, including individual, household, social, and demographic characteristics, are explained as follows:

The study looked at women aged 15 to 49 years old, who were categorized into three age groups: “15-29,” “30-39,” and “40-49” years; Years of schooling were categorized into “No Schooling” (0 years of education); “1-5 years” (5 years of schooling); “6-9” (6–9 years of schooling); and “10 and above” (10 and above years of schooling). The area of residence was categorized as “urban” and “rural”. Religion is classified as “Hindu”, “Muslim”, and “Others”. Caste was categorized as “SC/ST” (Scheduled Caste/Scheduled Tribes), “OBC” (Other backward classes), and “Others”. Children ever born were categorized into “0” (no child) “1” (one child ever born), “2” (two children ever born), “3” (three children ever born), and “4 and above” (four and more children ever born). Ever used a family planning method was categorized as “yes” (if ever used a family planning method) and “no” (for not used). The wealth quintile was classified as “Poorest”, “Poorer”, “Middle”, “Richer”, and “Richest”. Woman covered by Health insurance was categorized as “yes” and “no”. States taken into consideration are “Andhra Pradesh”, “Telangana”, and “Bihar”.

### Statistical analysis

#### Prevalence and binary logistic regression

Bivariate analysis has been utilized to understand the changes in the prevalence of hysterectomy among women aged 15-49 years by their socioeconomic and demographic variables and state of residence that is, in Andhra Pradesh, Telangana, and Bihar. The study has used logistic regression to ascertain the effect of various predictors on the outcome variable along with predicted probabilities [33]. The study population has adopted a multilevel approach to address the variability in the prevalence of hysterectomy due to various levels (i.e., at household, PSUs, and districts) in the study population. Fairlie decomposition has been computed to assess the contribution of various factors to the variation in the prevalence of hysterectomy in Andhra Pradesh, Telangana, and Bihar over two-time points.

The logistic regression analysis to examine the adjusted effects of various independent variables on hysterectomy among women aged 15–49 years used can be written as follows:

1 $$logit\left(P\left(Y=1|{x}_1,{x}_2,\dots, {x}_n\right)\right)={\beta}_0+{\beta}_1{x}_1+\dots +{\beta}_n{x}_n$$

Where, *β*_0_, *β*_1_, …, *β*_*n*_ are regression coefficients with Y being the response variable and *x*_*i*_^′^*s* being the predictor variables.

#### Predicted probabilities and Fairlie decomposition analysis

Based on the estimated model, predicted probabilities have been computed for both NFHS-4 and NFHS-5, followed by calculating the percent difference between the two predicted probabilities. Fairlie decomposition has been used to quantify the contributions of various risk factors to the difference in the predicted probabilities of hysterectomy by each factor from NFHS-4 to NFHS-5. It can be expressed as follows:

2 $${\overline{Y}}_u-{\overline{Y}}_r=\left[\sum\nolimits_{i=1}^{N^u}\frac{F\left({X}_i^u{\beta}^u\right)}{N^u}-\sum\nolimits_{i=1}^{N^r}\frac{F\left({X}_i^r{\beta}^u\right)}{N^r}\right]+\left[\sum\nolimits_{i=1}^{N^r}\frac{F\left({X}_i^r{\beta}^u\right)}{N^u}-\sum\nolimits_{i=1}^{N^r}\frac{F\left({X}_i^r{\beta}^r\right)}{N^r}\right]$$

Where, ${\overline{Y}}_u$ and ${\overline{Y}}_r$ represent mean value of hysterectomy at two-time points ‘u’ (2015-16) and ‘r’ (2019-20), ‘X’ represents the set of predictor variables, *β* represents the coefficient, *N*^*u*^ and *N*^*r*^ represent the sample size at time points u and r, respectively. First term in the equation represents characteristics and the latter represents the discrimination effect, the differences caused by various characteristic regression coefficients. A positive coefficient represents a positive contribution to the difference and vice-versa [12].

#### Multilevel regression analysis

A three-level logistic regression model was applied. The application of the multilevel modelling was justified by the hierarchal structure of the survey, where women were nested within households, the households were nested within PSUs and PSUs were nested within districts.

Three-level random intercept logistic model has been specified for the probability of an individual i in PSU j and district k having undergone the surgery (Y_ijk_ = 1)

3 $$\textrm{Logit}\ \left({\uppi}_{\textrm{ijk}}\right)={\upbeta}_{\textrm{o}}+{\upbeta \textrm{X}}_{\textrm{ijk}}+\left({\textrm{f}}_{0\textrm{k}}+{\textrm{v}}_{0\textrm{jk}}+{\textrm{u}}_{0\textrm{ij}}\right)$$

This model estimates the log odds of πijk adjusted for vector (X_ijk_) of above-mentioned independent variables measured at the individual level. The parameter βo represents the log odds of hysterectomy for a woman in the reference category of all the categorical variables. The random effect inside the brackets is interpreted as a residual differential for the district k (f_0k_), PSU j (v_0jk_) and individual i (u_0ij_) assumed to be independent and normally distributed with mean 0 and variance ${\sigma}_{f_0}^2$, ${\sigma}_{v_0}^2$, and ${\sigma}_{u_0}^2$, respectively. Finally, variance partitioning (measured by intra-class correlation coefficients (ICC)) quantifies the contribution by each level (i.e., HH, district, and PSU) to the total explained variation, in the log odds of women with hysterectomy [34].

## Results

**Fig. 1:**
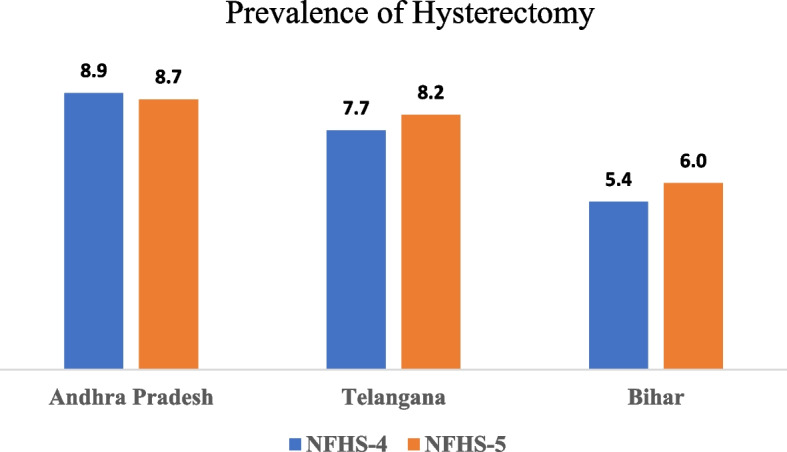
Prevalence of hysterectomy

Figure 1 shows that the prevalence of hysterectomy decreased from 8.9 to 8.7% in Andhra Pradesh but increased from 7.7 to 8.2% in Telangana and from 5.4 to 6% in Bihar.

The percentages of women aged 15-49 years who had a hysterectomy and the binary logistic regression odds ratio of the associated factors of hysterectomy in three high prevalence states of India in NFHS-4 (2015-16) and NFHS-5 (2019-21) are presented in Supplementary Tables 1A and 1B.

Table 1 depicts the difference in predicted probabilities of hysterectomy by years of schooling, place of residence, and wealth index in Andhra Pradesh, Telangana, and Bihar at two time points. It can be inferred that in Andhra Pradesh, the probability of hysterectomy increased by 1.4, 10.1, and 25.6% among those with no schooling, women with 6-9 years of schooling, and 10 and above years of schooling but decreased by 18.7% among women with 1-5 years of schooling, respectively. Similarly, in Telangana, the probability of hysterectomy among women decreased by 1.6 and 15.8% among women with no schooling and 1-5 years of schooling while it increased by 24.4, and 30.8% in women with 6-9 years of schooling, and 10 and above years of schooling, respectively. In Bihar, the predicted probability of hysterectomy increased for women with no schooling by 32.1%, for 1-5 years of schooling by 9.3% and 6-9 years of schooling by 3.5% but deceased for 10 and above years of schooling by 2.1%. Women living in rural areas show an increased probability of hysterectomy over time with 3.9 and 22.4% in Andhra Pradesh and Bihar, respectively, while in Telangana the probability of hysterectomy increased among women living in urban areas by 2.4%. Women belonging to the poorest and poorer wealth quintile in Andhra Pradesh show an increase in probability by 77 and 30.9%, respectively. In Telangana, women belonging to poorest, poorer, and middle wealth quintile show an increase by 14.9, 24.5, and 18.1%, respectively. Similarly, in Bihar, the probability of hysterectomy increased for women belonging to all the wealth quintiles except for women belonging to the poorest wealth quintile.

**Table 1:** Predicted probabilities of hysterectomy

	2015-16	2019-21	Difference
Andhra Pradesh	a	b	(b-a)/b*100
Years of schooling			
No schooling	0.123	0.124	1.4
1-5 years	0.126	0.102	−18.7
6-9 years	0.110	0.121	10.1
10 and above years	0.056	0.071	25.6
Residence			
Urban	0.090	0.076	−15.6
Rural	0.118	0.122	3.9
Wealth index			
Poorest	0.048	0.084	77.0
Poorer	0.088	0.115	30.9
Middle	0.107	0.096	10.4
Richer	0.114	0.125	9.5
Richest	0.145	0.128	−11.7
Telangana			
Years of schooling			
No schooling	0.143	0.141	−1.6
1-5 years	0.144	0.121	−15.8
6-9 years	0.077	0.096	24.4
10 and above years	0.039	0.051	30.8
Residence			
Urban	0.078	0.080	2.4
Rural	0.130	0.125	−3.4
Wealth index			
Poorest	0.067	0.077	14.9
Poorer	0.092	0.114	24.5
Middle	0.107	0.126	18.1
Richer	0.144	0.136	−5.4
Richest	0.155	0.155	−0.4
Bihar			
Years of schooling			
No schooling	0.071	0.094	32.1
1-5 years	0.077	0.084	9.3
6-9 years	0.071	0.073	3.5
10 and above years	0.052	0.051	−2.1
Residence			
Urban	0.064	0.068	6.3
Rural	0.069	0.085	22.4
Wealth index			
Poorest	0.054	0.054	−0.6
Poorer	0.075	0.076	1.2
Middle	0.090	0.092	2.2
Richer	0.094	0.101	7.2
Richest	0.099	0.099	0.1

The results of Fairlie decomposition to assess the contribution of various factors to the variation in the prevalence of hysterectomies in Andhra Pradesh, Telangana, and Bihar are presented in Table 2. From the results, the contributions of the explained variation differ substantially for the different measures of hysterectomy: 92.6% in Andhra Pradesh, 14.1% in Telangana, and 28.3% in Bihar. In Andhra Pradesh, the largest positive contributors to the difference are years of schooling and wealth index in the prevalence of hysterectomy whereas negative contributors are age and caste. In Telangana, the major contributors are age and place of residence, whereas previous use of a family planning method, and wealth index contribute to narrowing the gap. Lastly, in Bihar, the major positive contributor is age and wealth index, and to some extent health insurance status, while previous use of a family planning method acts as catalyst in narrowing down the gap between the prevalence of hysterectomy over two time points.

**Table 2:** Fairlie decomposition of hysterectomy in three high prevalence states of India

	**Andhra Pradesh**		**Telangana**		**Bihar**	
Number of obs.	16,368		26,177		62,098	
Difference	0.0026		−0.0051		−0.0112	
Total explained	0.0033		0.0007		0.0032	
% Total explained contribution	−92.6		−14.1		−28.3	
**Variable**	**Coeff.**	**% Contribution**	**Coeff.**	**% Contribution**	**Coef.**	**% Contribution**
Age	−0.0080	− 309.3	− 0.0196	381.6	− 0.0041	36.7
Schooling	0.0031	121.6	0.0014	−26.9	0.0006	−4.1
Place of residence	−0.0006	−21.8	− 0.0064	126.5	0.0001	−0.5
Religion	0.0001	−3.3	−0.0000	0.7	−0.0006	5.8
Caste	−0.0007	− 27.8	− 0.0013	24.8	0.0006	−5.5
Children ever born	−0.0009	−34.0	0.0000	0.9	−0.0008	6.9
Ever use of family planning	0.0004	14.1	0.0091	− 179.6	0.0192	− 170.5
Wealth index	0.0036	141.4	0.0053	− 104.6	0.0003	−2.7
Health insurance	0.0036	−18.9	0.0001	−2.5	−0.0002	1.5

Table 3 presents the multilevel logistic regression odds ratio of hysterectomy among women aged 15-49 years in three high prevalence states in India: Andhra Pradesh, Telangana, and Bihar in the years 2019-21.

**Table 3:** Multilevel logistic regression odds ratio of hysterectomy among women aged 15-49 years in three high prevalence states of India 2019-21

Background Characteristics	Andhra Pradesh Odds ratio (CI 95%)	Telangana Odds ratio (CI 95%)	Bihar Odds ratio (CI 95%)
Age (years)			
15-29			
30-39	5.89***(4.94,7.02)	8.64***(6.18,12.09)	5.89***(4.94, 7.02)
40-49	10.17***(8.49,12.18)	22.81***(14.70,35.40)	10.36***(8.65, 12.18)
Years of schooling			
No schooling			
1-5 years	0.88 (0.76,1.02)	0.77**(0.64,0.92)	0.88 (0.76,1.02)
6-9 years	0.74***(0.64,0.86)	0.55***(0.45,0.67)	0.74***(0.64,0.86)
10 and above years	0.48***(0.41,0.57)	0.26***(0.19,0.34)	0.48***(0.41,0.57)
Residence			
Urban			
Rural	1.35**(1.12,1.61)	1.76***(1.45,2.13)	1.35***(1.12,1.61)
Religion			
Hindu			
Muslim	0.72***(0.61,0.84)	0.50***(0.38,0.66)	0.72***(0.61,0.84)
Others	0.88 (0.25,3.06)	0.73 (0.51,1.03)	0.88 (0.25,3.06)
Caste/Tribe			
SC/ST			
OBC	1.14*(1.03,1.27)	1.15*(1.02,1.31)	1.14*(1.03,1.27)
Others	1.13 (0.97,1.32)	1.14 (0.92,1.40)	1.13 (0.97,1.32)
Children ever born			
1			
2	3.12***(2.27,4.29)	1.83***(1.46,2.30)	3.12***(2.27,4.29)
3	4.01***(2.93,5.49)	2.06***(1.62,2.62)	4.01***(2.93,5.49)
4+	3.69***(2.70,5.03)	1.84***(1.42,2.39)	3.69***(2.70,5.03)
Ever use of family planning			
No			
Yes	0.48***(0.43,0.54)	0.58***(0.49,0.69)	0.48***(0.43,0.54)
Wealth index			
Poorest			
Poorer	1.51***(1.29,1.75)	1.54***(1.29,1.83)	1.50***(1.29,1.75)
Middle	1.92***(1.65,2.23)	1.78***(1.47,2.17)	1.92***(1.65,2.23)
Richer	2.12***(1.81,2.49)	1.96***(1.58,2.43)	2.12***(1.81,2.49)
Richest	2.18***(1.81,2.63)	2.57***(1.94,3.42)	2.18***(1.81,2.63)
Health insurance			
No			
Yes	1.21**(1.07,1.38)	1.05 (0.93,1.20)	1.21**(1.07,1.38)
Constant	0.00***(0,00.01)	0.004***(0,0.01)	0.004***(0.00,0.01)
Var (district)	0.215	0.178	0.172
Var (PSU)	0.147	0.125	0.152
Var (HHs)	0.001	0.764	0.001
ICC (district) (%)	4.75	4.08	4.8
ICC (PSU) (%)	9.0	6.96	9.0
ICC (HHs) (%)	9.0	24.5	9.0

***p-value<0.01; **p-value<0.05; *p-value<0.10

Andhra Pradesh: Compared to women in the age group 15-29 years, women in the age group 30-39 years had 5.89 times and women in the age group 40 years and above had 10.17 times odds of having a hysterectomy. Compared to no schooling, 1-5 years of schooling, 10 years and above schooling, and having ever used a family planning method had a protective effect. Women living in rural areas, belonging to other religions, OBC and Other castes, who had two or three children, and in higher wealth quintiles had greater odds of having a hysterectomy than women living in urban areas, belonging to Hindu, SC/ST, having one child, and belonging to the poorest wealth quintile. Variation in the prevalence of hysterectomies among women is highest at the district level (σ^2^_district_ = 0.215), followed by PSU level (σ^2^_PSU_ = 0.147). ICC values show that about 4.8, 9.0, and 9.0% of the total variation in the prevalence of hysterectomies is attributed to the differences at district, PSU, and HH levels, respectively.

Telangana: Compared to women in the age group 15-29 years, women in the age group 30-39 years had 8.64 times and women in the age group 40 years and above had 22.81 times odds of having a hysterectomy. Compared to no schooling, 1-5 years of schooling, 6-9 years of schooling, 10 years and above schooling, being a Muslim, and having ever used a family planning method had a protective effect. Women living in rural areas, belonging to OBC, who had two or three or four and above children, and belonging to higher wealth quintiles had greater odds of having a hysterectomy than women living in urban areas, belonging to SC/ST, having one child, and belonging to the poorest wealth quintile. Variation in the prevalence of hysterectomies is highest at the HH level (σ^2^_HH_ = 0.764) followed by district level (σ^2^_district_ = 0.178) and at the PSU level (σ^2^_PSU_ = 0.125). ICC values show that about 24.5, 6.96, and 4.1% of the total variation in the prevalence of hysterectomies is attributed to the differences at HH, PSU, and district levels, respectively.

Bihar: Compared to women in the age group 15-29 years, women in the age group 30-39 years had 5.89 times and women in the age group 40 years and above had 10.36 times higher odds of having a hysterectomy. Compared to no schooling, 6-9 years of schooling, 10 years and above schooling, being a Muslim, and having ever used a family planning method had a protective effect. Women living in rural areas, belonging to OBC, who had two or more children, and in higher wealth quintiles had greater odds of having a hysterectomy than women living in urban areas, belonging to SC/ST, having one child, and belonging to the poorest wealth quintile. Variation in the prevalence of hysterectomies is highest at the district level (σ^2^_district_ = 0.175) followed by PSU level (σ^2^_PSU_ = 0.154) and at the HH level (σ^2^_HH_ = 0.001). ICC values show that about 9.0% of the total variation in the prevalence of hysterectomies is attributed to the differences at HH and PSU levels each, and 4.8% at the district level.

**Fig. 2:**
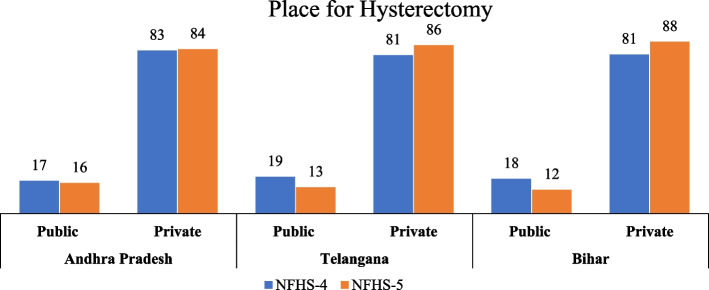
Place for hysterectomy

Figure 2 shows the bar graph representing the proportion of women getting hysterectomy done by place (i.e., public or private facilities) in each state at two time points (that is, NFHS-4 (2015-16) and NFHS-5 (2019-21)). The bar graph shows that the proportions of women who had hysterectomies in public facilities have decreased slightly in each of the states between the two time points. However, from NFHS-4 to NFHS-5, the proportion of women having hysterectomy in private hospitals has grown slightly, with 83 to 84% in Andhra Pradesh, 81 to 86% in Telangana, and 81 to 88% in Bihar, respectively. It is further noted that private facilities perform a higher number of hysterectomies than public facilities.

Further, hysterectomies due to single and multiple reasons among the study participants in all the three states can be seen in Table 4. It was found that excessive menstrual bleeding/pain and fibroids was the most prevalent reason due to which respondents opted for hysterectomies in all three states. However, the second most common pair of reasons for hysterectomy in Andhra Pradesh was excessive menstrual bleeding/pain and cervical discharge, whereas excessive menstrual bleeding/pain and uterine disorder was the second most common pair of reasons for hysterectomy in Telangana and Bihar. Figs. 1a, 1b and 1c in Supplementary Files depict the change in proportion for each reason between the two time points for each of the states. We did not include cervical discharge in the figures because NFHS-4 did not collect data on cervical discharge as a reason for hysterectomy.

**Table 4:** Proportion of hysterectomies due to single or multiple reasons in all three states, NFHS 5

	Andhra Pradesh	Telangana	Bihar
**Single Reason**			
Excessive menstrual bleeding/pain	33.53	36.77	43.66
Fibroids/cysts	19.93	32.78	6.38
Uterine disorder	2.04	4.97	7.69
Severe post-partum hemorrhage	1.79	1.39	1.63
Cervical discharge	3.68	2.15	3.4
Uterine prolapse	3.28	2.23	7.02
Cancer	1.13	1.04	4.51
Other	1.81	1.36	7.12
**Multiple reasons**			
Excessive menstrual bleeding/pain + Fibroids/cysts	16.14	9.03	3.42
Excessive menstrual bleeding/pain + Uterine disorder	0.9	2.2	3.13
Fibroids/cysts + Uterine disorder	0.66	1.28	0.37
Excessive menstrual bleeding/pain + Fibroids/cysts + Uterine disorder	0.46	0.22	1.6
Excessive menstrual bleeding/pain + Cancer	0.44	0.15	1.42
Excessive menstrual bleeding/pain + Cancer + Fibroids/cysts	0.11	0.03	0.03
Excessive menstrual bleeding/pain + Uterine prolapse	0.54	0.2	2.06
Excessive menstrual bleeding/pain + Cervical discharge	7.28	0.59	1.53
Fibroids/cysts + Cervical discharge	2.69	0.59	0.03

## Discussion

The current study is the first study to focus on the prevalence of hysterectomy and associated factors in the three high prevalence states of Andhra Pradesh, Telangana, and Bihar. While the national average for prevalence of hysterectomy is around 3%, in these three states it is over 6% (See supplementary Table 1A). According to a study based on women above 45 years of age, the prevalence was 11.4% with higher odds for women living in urban areas in wealthier indices. The highest prevalence (above 20%) was found in Andhra Pradesh and Punjab [2, 33]. As age increases the prevalence rates increase. However, a number of case studies document that hysterectomies are taking place among women under 30 years of age [13, 18, 25].

The predicted probabilities for years of schooling, place of residence and wealth index showed an increase from NFHS-4 to NFHS-5. The national average for women’s literacy, described as women “who completed Standard 9 or higher and women who can read a whole sentence or part of sentence”, is 71.5% with a higher average of 83% in urban areas and 65.9% in rural areas. In Andhra Pradesh, women’s literacy was 66.7%, in Telangana, it was 64.8% and in Bihar, it was 55% only. In rural areas, women’s literacy was even lower with 62, 56.6 and 51.6% in Andhra Pradesh, Telangana and Bihar, respectively. Women who had 10 years or above schooling were just 39.6, 45.5 and 28.8% in Andhra Pradesh, Telangana and Bihar, respectively [30]. These gaps in women’s literacy rates show up in terms of the percentage increase in predicted probabilities for hysterectomy in the three states. The percentage increase was higher among the more educated women in Andhra Pradesh and Telangana while it was highest among women with no schooling in Bihar. This shows that while higher women’s literacy was driving up the hysterectomy prevalence in Andhra Pradesh and Telangana, it was being driven up in Bihar by low literacy rates. This means that women with no schooling was the most vulnerable group in Bihar. Uneducated women remain prone to taking the doctor’s advice at face value and may not have enough exposure to seek counselling regarding alternate treatments. This is borne true in literature as well [16, 17, 22, 34, 35]. An in-depth analyses also revealed that women in rural areas and with no schooling were having hysterectomies done at a lower median age than women in urban areas and with higher education level [27]. On the other hand, women with higher literacy level may have higher perceived health literacy, and hence, may approach and utilize health services more often [36]. This is in evidence in the states of Andhra Pradesh and Telangana. However, this is a matter of concern because it shows that even educated women are not aware of less invasive alternative treatments for their conditions.

In Andhra Pradesh, the predicted probabilities increased in rural areas, in Telangana in urban areas and in Bihar in both urban and rural areas over the two time points. This may be attributed to the differing health outreach initiatives in the three states. This means that women in Bihar remained most vulnerable out of the three states as the pace at which it is increasing among rural women was the highest even though the overall probabilities were lower than Andhra Pradesh and Telangana. Our literature review revealed that in some studies women in rural areas had higher chances of having hysterectomy done [22, 24, 37] while in some other studies, women in urban areas had higher chances of getting hysterectomy done [2, 25, 33]. The difference may be attributed to factors other than place of residence such as education, socio-economic status and/ or availability of health services.

In Andhra Pradesh, women in poorest wealth quintile remained most vulnerable to have hysterectomy, while in Telangana the most vulnerable women belonged to the poorer wealth quintile, and in Bihar, it was women in the richer wealth quintile. A study done in Gujarat suggested that women in poorer and poorest wealth quintiles were vulnerable to choosing hysterectomies because of lack of awareness of reproductive issues and accessibility to health care facilities [32]. It also highlighted that the choice of hysterectomy was driven by women fearing loss of money either on alternative treatments, and/ or having to visit the health facility repeatedly. It may be conjectured that women in Bihar belonging to poorer and poorest wealth quintile remained less vulnerable for having hysterectomy because they were not be able to afford hysterectomies, thus, leaving women in higher wealth quintiles more likely to have access to the services. In contrast, women in poorest and poorer wealth quintiles in Andhra Pradesh and Telangana remained the most vulnerable group to choose hysterectomy due to issues of affordability of the surgery. Kumari and Kundu found that women having hysterectomies in poorer wealth quintile were 4 years younger than those in richer wealth quintiles [27]. Thus, it is important to look at factors locally to see if socially disadvantaged groups and individuals were choosing hysterectomy at a younger age as an option because it was less expensive in the long-run.

The Fairlie decomposition analyses revealed that the factor contributing positively to the variation in each of the three states was wealth index in Andhra Pradesh, age in Telangana and ever use of family planning method in Bihar. The overall percent total explained contribution was low in Telangana and Bihar which points out that there may be other factors or a combination of factors that contribute to the increase than those under study.

Multilevel logistic regression analyses revealed that the prevalence of hysterectomy increased with an increase in age and with increase in number of children ever born. Both these factors are known drivers of hysterectomy prevalence across the world. The chances of getting hysterectomy in the age group 30 to 39 years has increased substantially over the two time points compared to the reference category of women in 15 to 29 years [27]. This is a major concern since, hysterectomy before the age of 35 years is proven to have a negative impact on a woman’s physical and psychological wellbeing [24]. In all the three states, women living in rural areas, belonging to OBC, having two or more children, and belonging to higher wealth quintiles were more likely to have hysterectomy conducted compared to those women living in urban areas, belonging to SC/ST, having no or one child, and belonging to the poorest wealth quintile. This shows that women who consider their family to have reached a desirable level are more willing to have hysterectomy done than women who have one child. Literature review reveals a similar trend [17, 22, 26, 35]. However, in the study among women above 45 years of age, nulliparous women had the highest prevalence of hysterectomies compared with women with only one child though parity was not a significant factor [2]. Years of schooling and ever use of family planning method acted as protective factors in all the three states. This means that women with basic education levels are more pliant to using family planning methods and to take their decisions regarding their reproductive health than women with no schooling. This is consistent with other studies [17, 38]. Women living in rural areas are more prone to be worried about the cost of alternative remedies, cost of long-term medication, excessive bleeding and social and cultural taboos surrounding menstruation [15, 16, 27]. Age-specific studies have found lower age at first childbirth is also a contributing factor and it is more likely that women in rural areas undergo hysterectomies at a younger age because they were married early and had children at a younger age [17, 27, 35, 37]. A study on the costs of hysterectomy conducted in 2013 showed that the costs varied from 4124 rupees to 57,622 rupees [39]. This high variation in costs also needs to be investigated because it may affect a woman’s choice of treatment and her ability to pay. The low costs may outweigh the balance in favour of surgery than medication.

In our multilevel analyses, the ICCs for the hysterectomy was assigned at three levels: districts, PSUs, and HHs. HHs accounted for the highest ICC, indicating highest clustering in the prevalence of hysterectomy at HH level, followed by PSU, then district level in all the three states. This can mean that women may have a nearest-neighbor effect when considering undergoing the surgery based on another woman’s similar prior experiences that lead her to undergo the surgery. It also means that particular areas may have better health accessibility that drives the numbers up. These need to be looked into more carefully and an assessment has to be made if any private clinic or doctor is recommending the surgery with ulterior motives. Desai’s qualitative analyses of the experience of 35 women in Gujarat shows that women generally consulted two doctors before taking their decision based upon the long-term solutions. It is also driven by the fact that women themselves consider the womb/ uterus unnecessary beyond their child-bearing age and hence, once they consider that their families are complete, they opt for a more “permanent” solution to experiencing heavy menstrual bleeding and pain [14]. In these cases, it is an important finding that ever use of family planning method proved to be protective because those women were less likely to suffer from heavy bleeding and pain. It may also point to women’s greater awareness of seeking counselling services when needed. Desai et al. (2019) also found that state-level age specific factors for hysterectomy included having previous caesarean section, illiteracy and women’s employment while associating lower odds with sterilization [28]. A case study in Gujarat found sterilization to be associated with increased risk of hysterectomy [38]. About two-thirds of women utilized private hospitals while one third utilized state run hospitals and facilities.

**Fig. 3:**
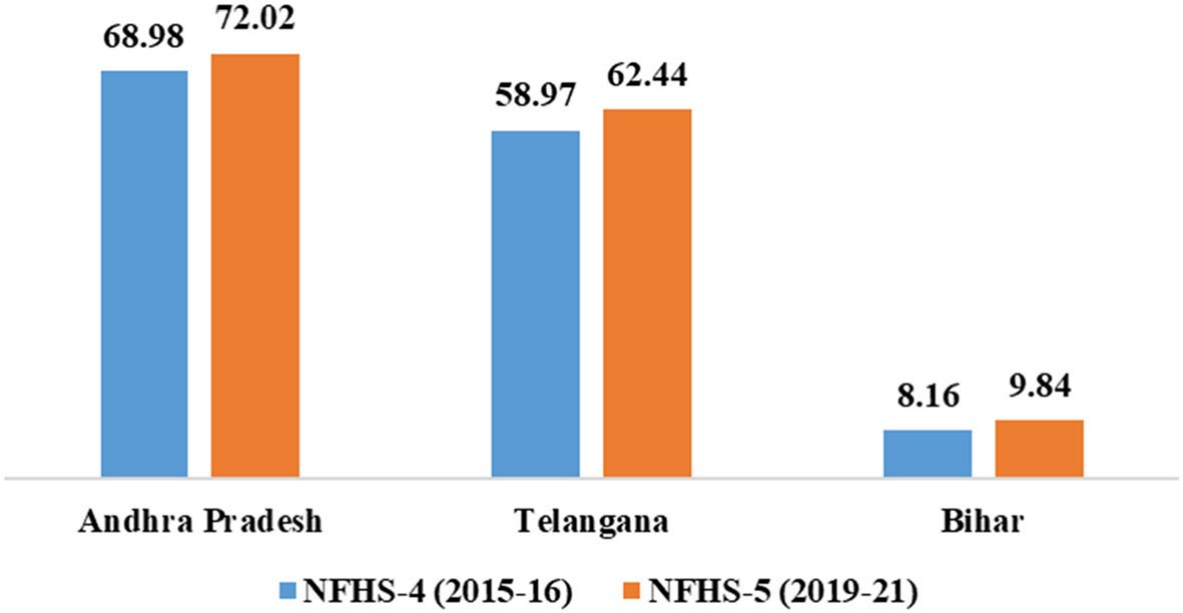
Proportions covered by health insurance over the last two rounds of NFHS survey

We also looked at whether more hysterectomies were conducted in private facilities than in public hospitals, and we found that more hysterectomies were conducted in the private facilities in both the rounds of NFHS. This shows that women may prefer private facilities because of ease and promptness provided by a private sector hospital [15]. However, it also makes women vulnerable to being advised for surgery due to lack of equipment, skills and/or knowledge of treatment options for less invasive options at private facilities [20, 28, 29]. Our analyses also reveals that the main reason for hysterectomy was heavy menstrual bleeding in all the three states. However, in Andhra Pradesh and Telangana, there was a sharp rise in surgeries due to fibroids/ cysts in NFHS-5 as compared to NFHS-4 which were also the most common pair of multiple reasons for hysterectomy in all the three states. In Bihar, there has been a rise in surgeries due to fibroids/ cysts and a dip in cases due to uterine disorder/prolapse. However, even if less invasive surgeries and pharmacological treatment as alternatives for hysterectomies are available or alternative options exist, as they might exist for women in urban areas in the richest and richer wealth scale, more hysterectomies may be taking place due to a doctor’s advice or because private hospitals may be using governmental health schemes for making money. It could also be that because hysterectomy is covered by many government-sponsored health insurance schemes, women are taking advantage of it [15–17]. From Fig. 3, clearly, there has been an increase in the proportion covered by health insurance in all three states. However, the insurance coverage is way higher in south Indian states of Andhra Pradesh and Telangana compared to Bihar.

In 2007, Aarogyasri health insurance program was introduced in Andhra Pradesh which aimed to provide underprivileged population with cashless health aids at tertiary healthcare. Since the programme provided higher reimbursement rates to hospitals than other comparable insurance plans, it was therefore identified as the cause of a sudden spike in hysterectomy rates in private facilities [16]. Corrective action was taken but it is still thought that the YSR Aarogyasri scheme in Andhra Pradesh may be driving the numbers up. In 2014, Telangana was carved out of Andhra Pradesh and a similar scheme remained in place. In 2015, Ayushman Bharat or Pradhan Mantri Jan Aroyga Yojana (PM-JAY) was introduced. The scheme is implemented in all three states. Supplemental Table 6 provides the change in proportions of women enrolled in various governmental and non-governmental insurance schemes over the two rounds of NFHS. In Andhra Pradesh and Telangana, the states have collaborated with the national scheme to make it more robust. Bihar did not have a state scheme prior to the introduction of the PM-JAY. While those covered by health insurance ranges between 60 to 70% in Andhra Pradesh and Telangana, it is less than 10% in Bihar. The wide gap in Bihar shows poor implementation of the PM-JAY and thus even though we should be worried about the high prevalence of hysterectomy in Andhra Pradesh and Telangana, agencies should focus more on Bihar to bring women into the schemes in order to ensure that they are provided health services as and when needed. Dubey et al., (2023) have shown the variation in utilization patterns of PM-JAY across all Indian states. They found that states with low poverty and disease burden utilize more services [40]. Hence, it is imperative to address the issue of increasing awareness and enrollment, particularly in the high prevalent state of Bihar.

## Limitations

Limitations of the study include the fact that it utilizes cross-sectional data of the survey, hence, no causal inferences can be made. Secondly, the present study worked with limited information regarding the medical history of the participants. For example, it was entirely possible that the women in this study who received a hysterectomy had tried several other medical options before opting for the surgery [41]. This study was unable to differentiate between medically necessary and “unnecessary” hysterectomies taking place in Andhra Pradesh, Telangana and Bihar. This study also did not take into account the provider’s preferences. It is often the case that the provider chooses to recommend hysterectomy rather than adopt evidence-based provider guidelines [29, 41–43]. Also, it was unclear whether health insurance cover was actually utilized for the hysterectomy or not. Lastly, since, hysterectomy was self-reported, therefore, it might have introduced a recall or reporting biases in the estimates.

## Conclusion and recommendations

Since the prevalence remained high in the three states in NFHS-4 and NFHS-5, we recommend better hysterectomy surveillance along with more efficient, accountable, and sustainable women health services. Gynaecological services beyond the child-bearing age need to be strengthened. Service providers must make sure that alternative treatments which are less invasive are accessible, available and affordable at the same time. A qualitative study is needed to assess providers’ KAP (knowledge, attitude and practices) about alternative treatment options for hysterectomy being done for benign conditions. If, however, these options are available but not getting utilized, more research may be needed to understand why women are selecting hysterectomies over these other options and educating women about these options may be a better approach. Government should adopt and implement standard regulatory practices to prevent avoidable provider-driven hysterectomy. Moreover, informing primary care professionals about the long-term health effects of hysterectomy and promoting alternate uterine fibroids and heavy bleeding therapies should be promoted.

### Supplementary Information

**Additional file 1.**

## Data Availability

The study utilizes a secondary source of data which is available on request and is available in the public domain through: https://dhsprogram.com/data/dataset/India_Standard-DHS_2020.cfm?flag=1.
