# Supplementary material for: Key drivers of hysterectomy among women of reproductive age in three states in India: comparative evidence from NFHS-4 and NFHS-5
Source: BMC Womens Health. 2024 Feb 9;24:107. doi: 10.1186/s12905-024-02886-7 (PMC10854047; doi:10.1186/s12905-024-02886-7)
Supplement: Supplementary file 1 — Additional file 1. [file 12905_2024_2886_MOESM1_ESM.docx]

**Supplementary Material**

From the results of Table 1A, in all three states, i.e., Andhra Pradesh, Telangana and Bihar, the highest percentage of women who had a hysterectomy is among women aged 40 and above. Also, over the two rounds of NFHS, the percentage has increased in Bihar significantly from 14.5 in NFHS-4 to 17.2 in NFHS-5 and has remained almost unchanged in Andhra Pradesh and Telangana. The highest proportion of women who had a hysterectomy had no schooling irrespective of the time and location with Bihar showing a significant increase in numbers from 7.8 to 10.5 percent. Comparatively, more women living in rural areas go for hysterectomy than women living in urban areas with 10.0, 10.3, and 6.2 percent (NFHS-5) in Andhra Pradesh, Telangana, and Bihar, respectively. Distribution by religion and caste does not show any trend in the proportion of women undergoing the procedure. Irrespective of the time and location, the proportion of women undergoing the procedure increases with the number of children ever born. A significant proportion of women who underwent hysterectomy had three children, 15.2 percent and 15.0 percent in Andhra Pradesh and Telangana and 13 percent in Bihar had four or more children according to the NFHS-5 (2019-21). Compared to their counterparts, a significant number of women who have ever used a family planning method have undergone hysterectomy with 11.5, 11.3, and 7.7 percent in Andhra Pradesh, Telangana, and Bihar, respectively, according to the NFHS-5 (2019-21). Lastly, those covered by health insurance reported a higher burden of hysterectomy in all three states across the last two rounds of the survey. While looking at the total proportion of women who had a hysterectomy in all three states over time, in Andhra Pradesh the proportion has decreased from 8.9 percent (NFHS-4) to 8.7 percent (NFHS-5). Though the absolute number of women who have had hysterectomies has increased significantly over the period of four years. In Telangana, the proportion has gone up from 7.7 percent (NFHS-4) to 8.2 percent (NFHS-5). Similarly, in Bihar, the proportion has increased from 5.4 percent in (NFHS-4) to 6.0 percent in (NFHS-5).

| **Table 1A: Percentage of women aged 15-49 who had hysterectomy by selected background characteristics in three high prevalence states of India** | | | | | | |
| --- | --- | --- | --- | --- | --- | --- |
|  | **Andhra Pradesh (%)** | | **Telangana (%)** | | **Bihar (%)** | |
| **Background Characteristics** | **2015-16** | **2019-21** | **2015-16** | **2019-21** | **2015-16** | **2019-21** |
| **Age (years)** |  |  |  |  |  |  |
| 15-29 | 1.11 (1.06, 1.17) | 0.59 (0.59, 0.59) | 1.06 (1.01, 1.13) | 0.38 (0.38, 0.38) | 1.00 (0.96, 1.03) | 0.65 (0.65, 0.65) |
| 30-39 | 9.57 (9.38, 9.77) | 7.79 (7.79, 7.79) | 9.48 (9.24, 9.71) | 8.09 (8.09, 8.10) | 8.20 (8.06, 8.34) | 10.00 (10.00, 10.00) |
| 40-49 | 22.37 (22.07, 22.66) | 22.45 (22.45, 22.45) | 20.09 (19.74, 20.44) | 21.20 (21.20, 21.20) | 14.50 (14.29, 14.72) | 17.19 (17.19, 17.19) |
| **Years of schooling** |  |  |  |  |  |  |
| No schooling | 14.68 (14.47, 14.90) | 16.08 (16.08, 16.08) | 15.64 (15.37, 15.91) | 17.11 (17.11, 17.11) | 7.38 (7.28, 7.47) | 10.52 (10.52, 10.52) |
| 1-5 years | 12.25 (11.95, 12.57) | 10.82 (10.82, 10.82) | 13.85 (13.36, 14.35) | 11.90 (11.90, 11.90) | 5.84 (5.65, 6.03) | 6.33 (6.33, 6.33) |
| 6-9 years | 7.82 (7.60, 8.05) | 8.53 (8.53, 8.53) | 4.67 (4.45, 4.90) | 5.94 (5.94, 5.94) | 3.53 (3.42, 3.64) | 3.29 (3.29, 3.29) |
| 10 and above years | 2.45 (2.36, 2.55) | 2.82 (2.82, 2.82) | 1.67 (1.60, 1.76) | 1.87 (1.87, 1.87) | 2.45 (2.37, 2.54) | 1.96 (1.96, 1.96) |
| **Residence** |  |  |  |  |  |  |
| Urban | 7.27 (7.10, 7.43) | 5.81 (5.81, 5.81) | 5.01 (4.88, 5.14) | 4.55 (4.55, 4.55) | 5.15 (5.00, 5.31) | 4.76 (4.76, 4.76) |
| Rural | 9.67 (9.54, 9.79) | 10.02 (10.02, 10.02) | 10.25 (10.07, 10.43) | 10.32 (10.32, 10.32) | 5.39 (5.34, 5.45) | 6.24 (6.24, 6.25) |
| **Religion** |  |  |  |  |  |  |
| Hindu | 9.18 (9.07, 9.30) | 8.79 (8.79, 8.79) | 8.20 (8.07, 8.32) | 8.89 (8.89, 8.89) | 5.58 (5.52, 5.65) | 6.29 (6.29, 6.29) |
| Muslim | 5.07 (4.81, 5.35) | 5.88 (5.88, 5.89) | 4.09 (3.84, 4.35) | 3.03 (3.03, 3.03) | 4.18 (4.05, 4.31) | 4.41 (4.41, 4.41) |
| Others | 9.93 (9.56, 10.32) | 9.90 (9.90, 9.90) | 6.28 (5.76, 6.84) | 6.23 (6.23, 6.23) | 6.70 (5.08, 8.80) | 5.12 (5.12, 5.13) |
| **Caste/Tribe** |  |  |  |  |  |  |
| SC/ST | 7.75 (7.56, 7.94) | 7.25 (7.25, 7.250 | 9.07(8.84, 9.31) | 8.10 (8.10, 8.10) | 4.78 (4.67, 4.90) | 5.81 (5.81, 5.81) |
| OBC | 9.48 (9.34, 9.63) | 8.54 (8.54, 8.54) | 7.45 (7.31, 7.60) | 8.36 (8.36, 8.36) | 5.60 (5.52, 5.68) | 6.33 (6.33, 6.33) |
| Others | 8.93 (8.72, 9.15) | 10.75 (10.75, 10.75) | 6.23 (5.98, 6.49) | 7.33 (7.33, 7.33) | 5.27 (5.14, 5.41) | 5.29 (5.29, 5.29) |
| **Children ever born** |  |  |  |  |  |  |
| 1 | 4.54 (4.33, 4.76) | 6.82 (6.82, 6.82) | 3.69 (3.48, 3.92) | 4.64 (4.64, 4.64) | 1.36 (1.27, 1.46) | 1.31 (1.31, 1.31) |
| 2 | 10.72 (10.55, 10.89) | 10.63 (10.63, 10.63) | 9.08 (8.88, 9.29) | 9.76 (9.76, 9.76) | 4.66 (4.52, 4.80) | 4.74 (4.74, 4.74) |
| 3 | 16.65 (16.33, 16.97) | 15.19 (15.18, 15.19) | 16.28 (15.92, 16.65) | 15.73 (15.73, 15.73) | 8.91 (8.73, 9.09) | 9.37 (9.37, 9.37) |
| 4+ | 14.07 (13.61, 14.54) | 13.14 (13.13, 13.14) | 11.92 (11.48, 12.38) | 15.73 (15.72, 15.73) | 10.31 (10.17, 10.46) | 12.97 (12.97, 12.97) |
| **Ever use of family planning** | |  |  |  |  |  |
| No | 2.96 (2.86, 3.06) | 4.37 (4.37, 4.370 | 5.64 (5.50, 5.78) | 3.55 (3.55, 3.55) | 5.28 (5.21, 5.35) | 3.73 (3.73, 3.73) |
| Yes | 12.58 (12.43, 12.73) | 11.47 (11.47, 11.47) | 9.65 (9.48, 9.82) | 11.26 (11.26, 11.26) | 5.59 (5.47, 5.71) | 7.74 (7.74, 7.74) |
| **Wealth index** |  |  |  |  |  |  |
| Poorest | 3.92 (3.58, 4.29) | 7.27 (7.27, 7.27) | 6.70 (6.25, 7.18) | 7.19 (7.19, 7.19) | 4.74 (4.66, 4.82) | 4.75 (4.75, 4.75) |
| Poorer | 8.52 (8.26, 8.78) | 10.21 (10.21, 10.21) | 9.42 (9.11, 9.74) | 10.16 (10.16, 10.17) | 6.03 (5.86, 6.15) | 6.14 (6.14, 6.14) |
| Middle | 9.68 (9.50, 9.87) | 8.10 (8.10, 8.10) | 8.39 (8.17, 8.62) | 9.48 (9.48, 9.48) | 6.03 (5.86, 6.19) | 7.08 (7.08, 7.08) |
| Richer | 9.15 (8.97, 9.33) | 9.61 (9.61, 9.61) | 8.50 (8.29, 8.72) | 7.84 (7.84, 7.84) | 5.93 (5.73, 6.13) | 6.76 (6.76, 6.76) |
| Richest | 8.38 (8.15, 8.62) | 8.09 (8.09, 8.09) | 4.95 (4.76, 5.14) | 5.94 (5.93, 5.94) | 5.21 (4.92, 5.51) | 5.16 (5.15, 5.16) |
| **Health insurance** |  |  |  |  |  |  |
| No | 6.80 (6.80,6.81) | 4.58 (4.48,4.58) | 5.48 (5.48, 5.48) | 5.27 (5.27, 5.27) | 6.37 (6.37, 6.37) | 5.61 (5.61, 5.61) |
| Yes | 12.21 (12.20, 12.21) | 10.16 (10.15, 10.16) | 12.20 (12.20, 12.20) | 9.87 (9.87, 9.87) | 10.29 (10.29, 10.30) | 9.39 (9.38, 9.39) |
| **Total** | **8.9 (8.7, 9.1)** | **8.7 (8.7, 8.7)** | **7.7 (7.5, 7.9)** | **8.17 (8.16, 8.17)** | **5.4 (5.3, 5.4)** | **6.00 (6.00, 6.01)** |

Results from Table 1B, it can be inferred that in Andhra Pradesh, Telangana and Bihar, women aged 40-49 had higher odds of hysterectomy compared to women in the age group 15-29 years. Schooling, especially 10 years and above, and ever use of family planning methods act as protective factors. The odds of having a hysterectomy were higher for women living in rural areas, having two or more children and for women in the richest quintile of the wealth index. Women who have 10 and above years of schooling show 0.50 times lower risk of hysterectomy in Andhra Pradesh, 0.31 times lower risk in Telangana, and 0.31 times lower risk in Bihar as compared to those who have had no schooling (NFHS-5 (2019-21)). Women living in rural areas have higher odds of hysterectomy as compared to women living in urban areas with 1.80, 1.75, and 1.26 times the odds in Andhra Pradesh, Telangana, and Bihar, respectively in NFHS-5 (2019-21). Women who had two or more children ever born have 1.53 times higher odds of hysterectomy as compared to those with one child in Andhra Pradesh. Similarly, in Telangana, the risk increases with the number of children ever born as compared to those with one child. In Bihar, women with three children ever born have 3.9 times higher odds of hysterectomy than those with one child. Over time, Andhra Pradesh women who have ever used a family planning method show a 0.55 times lower risk of hysterectomy in NFHS-5 (2019-21). In Telangana, the risk is 0.66 times lower than for those who have never ever used a family planning method. Similarly, in Bihar, it is 0.48 times lower than their respective counterpart. Women with the richest wealth index have 2.4, 3.1 and 1.3 times higher odds of hysterectomy in Andhra Pradesh, Telangana, and Bihar, respectively. Lastly, those covered by health insurance have 1.3 times higher odds of hysterectomy each in Andhra Pradesh and Bihar compared to those not covered by health insurance. However, in Telangana, health insurance does not show any significant association with the prevalence of hysterectomy.

| **Table 1B: Binary logistic regression odds ratio of associated factors of hysterectomy among women aged 15-49 in three high prevalence states of India** | | | | | | |
| --- | --- | --- | --- | --- | --- | --- |
|  | **Andhra Pradesh Odds ratio (CI 95%)** | | **Telangana Odds ratio (CI 95%)** | | **Bihar Odds ratio**  **(CI 95%)** | |
| **Background Characteristics** | **2015-16** | **2019-21** | **2015-16** | **2019-21** | **2015-16** | **2019-21** |
| **Age (years)** |  |  |  |  |  |  |
| 15-29 |  |  |  |  |  |  |
| 30-39 | 4.46***(3.24,6.13) | 7.40***(4.91,11.16) | 3.83***(2.76,5.32) | 8.22***(6.14,11.00) | 3.80***(3.24,4.46) | 5.73***(4.82,6.82) |
| 40-49 | 11.31***(8.11,15.29) | 21.45***(14.27,32.25) | 7.51***(5.38,10.49) | 20.06***(14.99,26.85) | 6.62***(5.62,7.79) | 9.63***(8.06,11.51) |
| **Years of schooling** |  |  |  |  |  |  |
| No schooling |  |  |  |  |  |  |
| 1-5 years | 1.03(0.84,1.27) | 0.77*(0.63,0.95) | 1.00(0.77,1.32) | 0.82*(0.71,0.96) | 1.09(0.94,1.26) | 0.89(0.77,1.02) |
| 6-9 years | 0.88(0.70,1.10) | 0.94(0.76,1.16) | 0.47***(0.34,0.65) | 0.62***(0.53,0.72) | 1.00(0.86,1.16) | 0.77***(0.67,0.88) |
| 10 and above years | 0.41***(0.31,0.55) | 0.50***(0.39,0.65) | 0.23***(0.16,0.33) | 0.31***(0.26,0.37) | 0.7***(0.59,0.83) | 0.52***(0.44,0.61) |
| **Residence** |  |  |  |  |  |  |
| Urban |  |  |  |  |  |  |
| Rural | 1.37**(1.13,1.66) | 1.80***(1.48,2.20) | 1.84***(1.44,2.36) | 1.75***(1.53,2.00) | 1.09 (0.94,1.27) | 1.26**(1.07,1.48) |
| **Religion** |  |  |  |  |  |  |
| Hindu |  |  |  |  |  |  |
| Muslim | 0.55***(0.40,0.77) | 0.65**(0.47,0.89) | 0.72(0.49,1.06) | 0.52***(0.41,0.66) | 0.68***(0.59,0.79) | 0.64***(0.55,0.74) |
| Others | 1.32(0.97,1.78) | 1.77***(1.36,2.31) | 0.85(0.50,1.44) | 0.81(0.59,1.10) | 0.60(0.14,2.59) | 0.66(0.20,2.19) |
| **Caste/Tribe** |  |  |  |  |  |  |
| SC/ST |  |  |  |  |  |  |
| OBC | 1.34**(1.09,1.64) | 1.41**(1.15,1.73) | 0.81*(0.66,0.99) | 1.11(0.99,1.23) | 1.28***(1.14,1.44) | 1.18**(1.07,1.31) |
| Others | 1.01(0.79,1.30) | 1.61***(1.27,2.05) | 0.85(0.61,1.16) | 1.09(0.91,1.31) | 1.22*(1.04,1.42) | 1.16(0.99,1.34) |
| **Children ever born** |  |  |  |  |  |  |
| 1 |  |  |  |  |  |  |
| 2 | 1.35(0.97,1.88) | 1.53**(1.16,2.02) | 2.2***(1.51,3.20) | 1.68***(1.39,2.03) | 2.95***(2.19,3.98) | 3.08***(2.25,4.21) |
| 3 | 1.43*(1.02,2.02) | 1.51**(1.12,2.04) | 2.48***(1.69,3.65) | 1.77***(1.46,2.16) | 4.38***(3.27,5.87) | 3.91***(2.87,5.33) |
| 4+ | 0.90(0.61,1.33) | 1.10(0.76,1.59) | 1.35(0.88,2.07) | 1.50***(1.21,1.86) | 3.68***(2.75,4.93) | 3.61***(2.65,4.90) |
| **Ever use of family planning** | |  |  |  |  |  |
| No |  |  |  |  |  |  |
| Yes | 1.24(0.96,1.60) | 0.55***(0.44,0.68) | 0.53***(0.44,0.65) | 0.66***(0.58,0.76) | 0.50***(0.45,0.55) | 0.48***(0.43,0.53) |
| **Wealth index** |  |  |  |  |  |  |
| Poorest |  |  |  |  |  |  |
| Poorer | 2.00**(1.13,3.52) | 1.37(0.88,2.16) | 1.44(0.95,2.19) | 1.50**(1.16,1.93) | 1.43***(1.28,1.60) | 1.48***(1.33,1.65) |
| Middle | 2.53**(1.47,4.38) | 1.69*(1.09,2.62) | 1.74**(1.16,2.61) | 2.19***(1.71,2.81) | 1.79***(1.55,2.05) | 1.62***(1.42,1.84) |
| Richer | 2.76***(1.58,4.81) | 2.00**(1.28,3.14) | 2.59***(1.69,3.96) | 2.62***(2.03,3.38) | 1.92***(1.52,2.67) | 1.57***(1.31,1.87) |
| Richest | 3.76***(2.08,6.78) | 2.36**(1.45,3.87) | 2.93***(1.77,4.85) | 3.08***(2.30,4.11) | 2.02***(1.52,2.67) | 1.32(0.99,1.76) |
| **Health insurance** |  |  |  |  |  |  |
| No |  |  |  |  |  |  |
| Yes | 1.10(0.91,1.33) | 1.29*(1.04,1.61) | 1.16(0.94,1.44) | 1.06(0.95,1.18) | 1.49***(1.31,1.71) | 1.28***(1.13,1.44) |
| **Constant** | 0.004 | 0.004 | 0.013 | 0.004 | 0.004 | 0.006 |

***: p-value <0.001 level of significance; **: p-value<0.01 level of significance; *: p-value<0.05 level of significance.

**Figure 1a** shows the bar graph representing the proportion of women getting hysterectomies done due to various reasons (i.e., excessive menstrual bleeding/pain, fibroids/cyst, uterine disorder/prolapse, cancer, or severe post-partum hemorrhage) in Andhra Pradesh at two time points (i.e., NFHS-4 (2015-16) and NFHS-5 (2019-21)). In NFHS-4, the most common reason for hysterectomies was excessive menstrual bleeding/pain, which accounted for 64 percent of the cases, followed by fibroids/cysts, which accounted for 23 percent. However, in NFHS-5, though the number of women who had hysterectomies because of heavy monthly bleeding/pain remained high, 41 percent women cited fibroids/cysts as the reason. Although fibroids/cysts as a reason for hysterectomy among women increased from NFHS-4 to NFHS-5, uterine disorder/ prolapse showed a slight reduction. Cancer as a reason for hysterectomy accounts for only 2.6 percent of cases, whereas severe post-partum hemorrhage accounts for 3.7 percent in NFHS-5 (2019-21).

**Figure 1b** shows the bar graph representing the proportion of women getting hysterectomies done due to various reasons (i.e., excessive menstrual bleeding/pain, fibroids/cyst, uterine disorder/prolapse, cancer, or severe post-partum hemorrhage) in Telangana at two time points (i.e., NFHS-4 (2015-16) and NFHS-5 (2019-21)). The proportion of women who had hysterectomies done in NFHS-4 was primarily owing to heavy menstrual bleeding/pain with 48 percent, followed by fibroids/cysts with 34 percent. However, in NFHS-5, excessive menstrual bleeding/pain and fibroids/cyst are the two most common causes, accounting for 49 and 48 percent, respectively. Fibroids/cysts as a reason for hysterectomy has increased among women from NFHS-4 to NFHS-5. Meanwhile, the proportion of women with uterine disease or prolapse has decreased dramatically, from 26 percent in NFHS-4 to 11 percent in NFHS-5. In the NFHS-5 (2019-21) in Telangana, cancer, and severe post-partum hemorrhage account for the least number of hysterectomies, with 1.9 and 1.6 percent, respectively.

**Figure 1c** shows the bar graph representing the proportion of women getting hysterectomies done due to various reasons (i.e., excessive menstrual bleeding/pain, fibroids/cyst, uterine disorder/prolapse, cancer, or severe post-partum hemorrhage) in Bihar at two time points (i.e., NFHS-4 (2015-16) and NFHS-5 (2019-21)). The graph shows that in NFHS-4, the most common reason for hysterectomies was excessive menstrual bleeding/pain, which accounted for 54 percent, followed by uterine disorder/prolapse, which accounted for 34 percent in NFHS-4. In Bihar, the number of women undergoing hysterectomy because of severe menstrual bleeding/pain has risen to 58 percent. Uterine disorder/prolapse was also a major cause of hysterectomy at 25 percent, followed by fibroids/cysts at 13 percent, cancer at 6.8 percent and severe post-partum hemorrhage at 3.2 percent in NFHS-5.

Table 5 depicts the health insurance coverage across three states over the last two rounds in the study sample; it may be noted that there has been an increase in the proportion covered by health insurance in all three states. However, the insurance coverage was way higher in the South Indian states of Andhra Pradesh (72.0% (NFHS-5)) and Telangana (62.4% (NFHS-5)) than in Bihar (9.8% (NFHS-5)).

| **Table 5: Health Insurance coverage across three states over the last two rounds of NFHS** | | |
| --- | --- | --- |
| **States** | **Covered by Health Insurance** | |
|  | **NFHS-4 (2015-16)** | **NFHS-5 (2019-21)** |
| Andhra Pradesh | 68.98 (6059/8801) | 72.02 (8202/10975) |
| Telangana | 58.97 (3848/6179) | 62.44 (18078/27518) |
| Bihar | 8.16 (3061/36567) | 9.84 (4432/42483) |

Table 6 shows that the proportion covered under all types of health insurance has increased in all three states over the last two rounds of the survey. In Andhra Pradesh and Telangana, most women were covered under the “State Health Insurance Scheme”. However, in Bihar, most women were covered under the “Rashtriya Swasthya Bima Yojana”, with approximately 6.4 percent in NFHS-4. In NFHS-5, it was “Other Health Insurance,” under which most women in Bihar were covered with approximately 7.7 percent.

| **Table 6: Health insurance coverage by type across three states over the last two rounds of NFHS** | | | | | | |
| --- | --- | --- | --- | --- | --- | --- |
| **Insurance type** | **NFHS-4** | | | **NFHS-5** | | |
|  | **Andhra Pradesh** | **Telangana** | **Bihar** | **Andhra Pradesh** | **Telangana** | **Bihar** |
| **Employees State Insurance Scheme (ESIS)** | 1.27 (117) | 2.05 (99) | 0.27 (87) | 1.55 (154) | 2.22 (500) | 0.15 (57) |
| **Central Government Health Scheme (CGHS)** | 0.42 (33) | 0.74 (43) | 0.69 (203) | 2.28 (258) | 0.44 (93) | 0.90 (365) |
| **State Health Insurance Scheme** | 67.18 (8801) | 54.46 (3605) | 0.61 (173) | 70.53 (7889) | 59.81 (17372) | 0.09 (43) |
| **Rashtriya Swasthya Bima Yojana** | 0.02 (2) | 1.11 (80) | 6.39 (2539) | 0.83 (76) | 0.05 (12) | 1.63 (739) |
| **Community Health Insurance Programme** | 0.08 (6) | 0.07 (7) | 0.09 (25) | 0.06 (5) | 0.07 (16) | 0.04 (16) |
| **Other Health Insurance Through Employer** | 0.13 (12) | 0.39 (25) | 0.03 (12) | 0.13 (14) | 0.33 (74) | 0.02 (8) |
| **Medical Reimbursement from Employer** | 0.19 (18) | 0.25 (16) | 0.05 (14) | 0.17 (16) | 0.14 (32) | 0.03 (11) |
| **Other Privately Purchased Commercial Health Insurance** | 0.32 (25) | 0.88 (32) | 0.06 (15) | 1.02 (101) | 0.51 (99) | 0.06 (27) |
| **Other** | 0.05 (4) | 0.11 (7) | 0.04 (11) | 1.57 (143) | 0.23 (65) | 7.72 (3213) |
